# Natural killer cells limit the clearance of senescent lung adenocarcinoma cells

**DOI:** 10.1038/s41389-019-0133-3

**Published:** 2019-04-01

**Authors:** Kate L. Stokes, Virna Cortez-Retamozo, Jonuelle Acosta, Brian Lauderback, Camila Robles-Oteiza, Michelle Cicchini, Mikael J. Pittet, David M. Feldser

**Affiliations:** 1 Department of Cancer Biology, University of Pennsylvania, 421 Curie Blvd., 751 BRB II/III, Philadelphia, PA 19104-6160 USA; 2 Center for Systems Biology, Massachusetts General Hospital Research Institute and Harvard Medical School, Boston, MA 02114 USA; 3 Cell and Molecular Biology Graduate Program, University of Pennsylvania, 421 Curie Blvd., 751 BRB II/III, Philadelphia, PA 19104-6160 USA; 4 Abramson Family Cancer Research Institute, University of Pennsylvania, 421 Curie Blvd., 751 BRB II/III, Philadelphia, PA 19104-6160 USA

## Abstract

Senescence is an important p53-controlled tumor suppressor program that not only opposes the proliferation of cancer cells but also promotes their immune-mediated clearance in certain contexts. In hepatocellular cancer, p53 induction promotes an innate immune cell-mediated clearance of senescent cells wherein natural killer (NK) cells seem to play the primary sentinel role. Whether NK cells also surveil cancer cells in other tumor types when p53 is activated to promote a senescence response is unknown. To identify the role that NK and other innate immune cell types have on the surveillance and destruction of lung adenocarcinoma cells, we developed an orthotopic transplantation model where p53 gene function could be restored to induce senescence after successful engraftment of tumor cells in the mouse lung. Contrary to precedent, we found that NK cells actually limited the efficient clearance of tumor cells from the mouse lung after p53 restoration. Instead, activation of p53 induced the infiltration of monocytes, neutrophils, and interstitial macrophages. Loss of NK cells further promoted expansion of these inflammatory cell types and tumor clearance after p53 restoration. These observations suggest that NK cell responses to p53 activation in lung adenocarcinoma is distinct from those found in other tumor types and that diverse innate immune cell populations may play context-dependent roles during tumor immune surveillance. Further, our data provide an impetus to understand the broader mechanisms that regulate cancer cell destruction by multiple cell types of the innate immune system and distinct cancer contexts.

## Introduction

The cancer immunoediting hypothesis posits that the immune system shapes the evolution of tumor cells toward a cellular state that is poorly recognized by the immune system^1^. Advanced tumors that have evolved over many cellular divisions therefore have been selected for cells that lack expression of potent antigens or promote a tumor microenvironment that shields cancer cells from immune detection or destruction^2^. More recently, it has become better appreciated that this latter effect is greatly impacted by the same mutations in common oncogenes and tumor suppressors that activate canonical cancer cell intrinsic mechanisms to drive initiation and progression of the disease^3–6^. Because mechanisms that foster immune evasion can be the same as those that mediate oncogenesis, reinstating tumor suppressive pathways in cancer cells may render established tumors vulnerable to immune-mediated destructive mechanisms, which can be harnessed for therapeutic gain.

The p53 tumor suppressor controls a diverse array of cellular programs that are induced in a context dependent manner to suppress or eradicate cancer^7^. Most commonly appreciated, activation of the p53 pathway can induce apoptosis, a form of mitochondria-associated caspase-dependent cell death that is in many cases considered to be non-immunogenic or even tolerogenic^8^. However, p53-induced cellular senescence is a major mechanism of tumor suppression that actively promotes immune responses^5,7,8^. In addition to irreversibly halting the cell cycle, cellular senescence also induces a secretory phenotype that in certain contexts recruits immune cells that ultimately carry out destruction of the senescent cancer cells and healing of the affected tissue site^9,10^. Genetically engineered mouse models, wherein a previously inactive p53 pathway can be toggled back on in established liver cancers in the mouse, have highlighted that subsequent to the induction of senescence, multiple cell types of the innate immune system infiltrate tumors in response to p53 reactivation and that natural killer (NK) cells play a key and direct role in destroying senescent liver cancer cells^11–13^. While the cellular and molecular determinants of p53-mediated tumor immune surveillance in hepatocellular carcinoma are only beginning to be uncovered, given the pleiotropic nature of p53-controlled responses, it is clear that identifying the precise cellular or molecular mechanisms that are involved in other tumor types is needed. These insights could aid in the development of cell-based or molecular therapies that mimic the effects of p53 reactivation at the level of the cancer cell or the microenvironment.

Previously, we modeled the effects of therapeutic reactivation of p53 in established mouse lung adenocarcinomas^14^. In the model, tumors are initiated by the spontaneously activating *Kras*^*LA2*^ allele that expresses KRAS^G12D^ after a rare and stochastic recombination event in somatic cells in the mouse lung^15^. We regulated p53 expression using the *Trp53*^*LSL*^ allele that harbors a floxed transcriptional *‘STOP’* cassette inserted within the first intron of the *Trp53* locus and a ubiquitously expressed *Rosa26*^*CreER*^ allele to control the timing of p53 reactivation via tamoxifen administration^16^. Despite efficient *Trp53* gene restoration in all tumor cells, the activation of p53-mediated tumor suppression occurred selectively only in high grade tumor cells, which were subsequently culled from the overall tumor mass. Culling of tumor cells was coincident with the induction of cell cycle arrest, features of cellular senescence, and the presence of immune cell infiltrates^14^. To better understand p53-mediated immune surveillance in this model, here we develop orthotopically transplantable lung adenocarcinoma cell lines from *Kras*^*LA2/+*^, *Trp53*^*LSL/LSL,*^*Rosa26*^*CreER/CreER*^ (KPr) tumors where the endogenous *Trp53* locus can be restored to a wild-type state after engraftment into the mouse lung. We demonstrate that p53 reactivation in KPr cells potently induces senescence and a complex inflammatory immune response in the lung involving multiple innate immune cell types including NK cells. However, we find that although NK cells display a marker of activation after p53 restoration, they surprisingly do not target tumor cells for destruction but instead act to effectively dampen the culling of lung adenocarcinoma cells from the lung. Loss of NK cells led to a greater influx of inflammatory immune cells and a more rapid and effective clearance of lung adenocarcinoma cells after p53 reactivation. That these observations contrast those observed in the context of liver cancer models, highlights the importance of cellular and tissue context in dictating the outcome of p53 actions in cancer cells.

## Results

### Reactivation of p53 induces senescence and tumor cell clearance in the absence of adaptive immunity

**Fig. 1:**
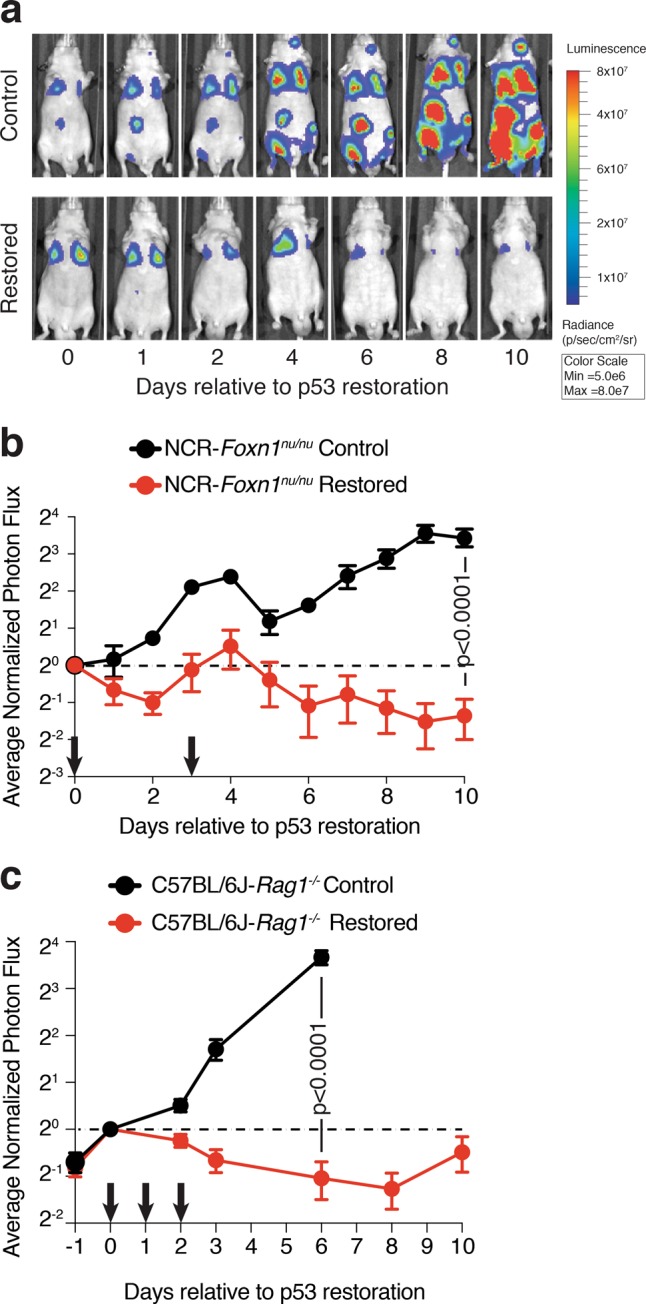
Restoration of p53 in orthotopically transplanted lung adenocarcinomas causes tumor regression in mice lacking adaptive immunity. **a** Representative bioluminescent optical imaging of *Foxn1*^*nu/nu*^ mice transplanted with KPrLG cells. The initial treatment with corn oil (Control) or tamoxifen (Restored) is defined as day 0. Subsequent treatments indicated by arrows on *x*-axis. Images, relative to day 0 are from the same animal at the indicated time points. **b** Quantification of bioluminescent images from NCR:*Foxn1*^*nu/nu*^ mice. Corn oil or tamoxifen was administered on day 0 and day 3. Data shown are combined from two independent KPr cell lines. **c** Quantification of bioluminescent images from C57BL6/J:*Rag1*^*-/-*^ mice. Corn oil or tamoxifen was administered on days 0, 1, and 2 (arrows). Analysis of significance between control and restored groups was performed by *t*-test at day 10 test (*Foxn1*^*nu/nu*^) day 6 (C57BL6/J:*Rag1*^*-/-*^)

We sought to develop an orthotopic lung transplantation approach to control the timing and extent of tumor growth in the mouse lung and ultimately determine the contribution of innate immune cell types to tumor clearance after p53 induction. We used multiple KPr cell lines that were established from individual lung adenocarcinomas growing in *Kras*^*LA2/+*^, *Trp53*^*LSL/LSL*^, *Rosa26*^*CreER/CreER*^ mice^14^. Exposing these cells to 4-hydroxytamoxifen in vitro strongly induces p53 expression and downstream target genes within 24 h^14^. Importantly, p53 induction does not lead to appreciable levels of cell death but instead induces a rapid cell cycle arrest that is followed by the onset of cellular senescence (14 and Figure S1). We modified KPr cells to express green fluorescent protein (GFP) and luciferase via retroviral transduction of an MSCV-driven luciferase-IRES-GFP construct (KPrLG). Intravenous injection of KPrLG cells into the tail vein of immunocompromised *Foxn1*^*Nu/Nu*^ (nude) mice results in robust tumor cell engraftment into the lungs of each recipient mouse that can be imaged via bioluminescence techniques (Fig. 1a). To determine the effect of p53 reactivation on the maintenance of established tumors, we treated cohorts of nude mice harboring KPrLG cells with tamoxifen dissolved in corn oil, or vehicle alone, after tumor establishment, which was defined by overcoming a threshold radiance level (p/sec/cm^2^/sr) (see methods). The initial exposure to tamoxifen was designated as day 0 relative to p53 restoration. Treatment with tamoxifen on day 0 and again on day 3 to reactivate p53 resulted in significant cessation of tumor growth and regression of tumors over a 10 day monitoring period (Fig. 1a, b). By day 10, luminescence values had decreased to ~25% of that originally observed at day 0 in mice after p53 restoration, whereas luminescence values in vehicle-treated control mice had increased greater than 300% (Fig. 1b). These data suggest that unlike the cytostatic effects of p53 reactivation that occur in vitro (Figure S1), when growing in the lung microenvironment, p53 reactivation also results in the elimination of a significant fraction of KPrLG cells from the lung. The loss of KPrLG cells after p53 reactivation when engrafted in the lungs of nude mice that have a severely compromised adaptive immune system suggests that cells from the B and T cell lineages are not required for tumor regression. To more stringently test that hypothesis, we grafted KPrLG cells into the lungs of C57BL/6 J: *Rag1*^*-/-*^ mice that completely lack B and T cells. Again, reactivation of p53 expression via tamoxifen exposure on days 0, 1, and 2 resulted in significantly reduced tumor burden. By 8 days post-p53 reactivation greater than 50% of the luminescent signal was lost, and by 10 days some tumor cells that escaped Cre-mediated recombination of the *p53*^*LSL*^ allele began to regrow (Fig. 1c). These observations indicate that p53 reactivation in lung adenocarcinoma cells leads to tumor regression in vivo in the absence of B and T cells.

**Fig. 2:**
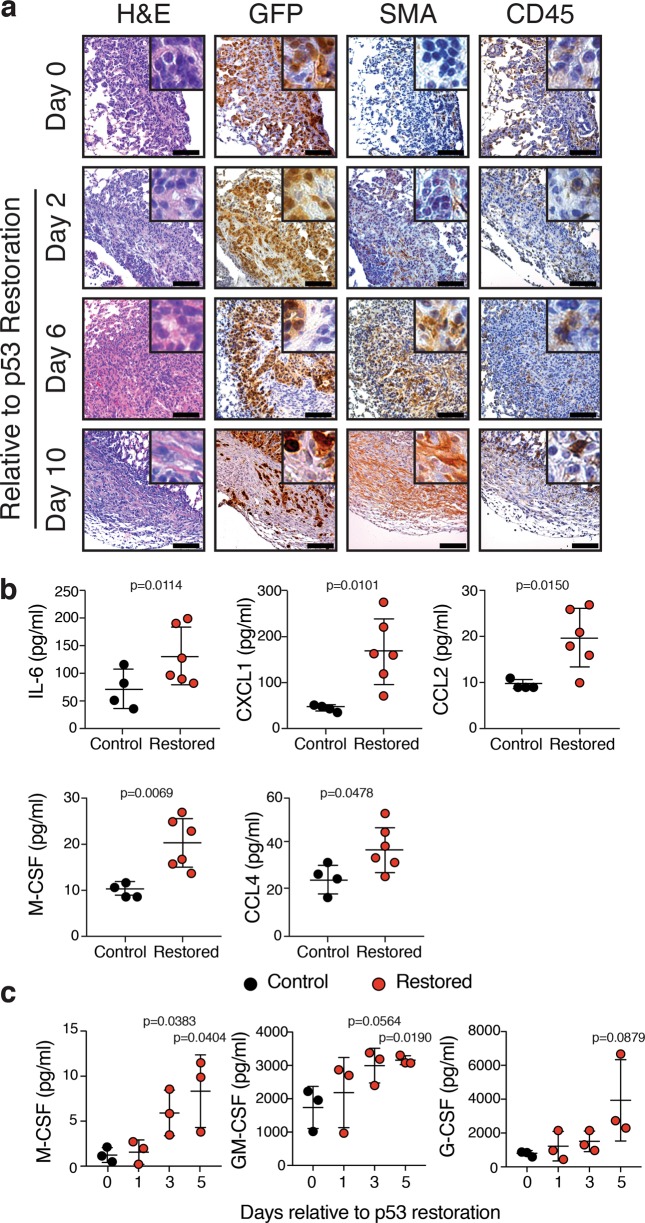
Restoration of p53 in orthotopically transplanted lung adenocarcinomas causes an infiltration of CD45^pos.^ immune cells and fibroblasts and elevated chemokine levels. **a** Histological sections from Control and p53-Restored tumors at Day 2, 6, and Day 10. H&E and IHC for GFP, CD45, and SMA. **b** Multiparameter cytokine detection in bronchioalveolar lavage (BAL) of Control and p53-Restored mice. Significantly changing cytokines as determined by *t*-test are indicated (IL-6, CCL2, CXCL1, M-CSF, and CCL4). **c** Multiparameter cytokine detection in KPrLG cell culture supernatant after p53 restoration in vitro

To investigate the response to p53 reactivation at the tissue level, we harvested lungs from *Rag1*^*-/-*^ mice that were transplanted with KPrLG cells and treated with vehicle or tamoxifen to reactivate p53. We assessed tissue sections to detect transplanted KPrLG tumor cells by immunohistochemistry for GFP and immune cells with the pan leukocyte marker CD45. In the absence of p53 reactivation, tumors growing in the lungs of *Rag1*^*-/-*^ mice were densely packed and homogeneously positive for GFP (Fig. 2a). In contrast, p53 reactivation led to tumor areas that were progressively less likely to be GFP positive but were infiltrated with increasing numbers of CD45-positive cells over time (Fig. 2a). Additionally, as the GFP-positive KPrLG cells receded, tumor areas also became infiltrated with smooth muscle actin (SMA)-positive fibroblasts (Fig. 2a). p53 directly and indirectly controls the expression of multiple cytokines and chemokines that could affect the recruitment or expansion of innate immune cells. Consistently, increased amounts of immune cell chemoattractants and inflammatory cytokines were present in bronchoalveolar lavage fluid of mice after p53 restoration that include IL-6, CXCL1, CCL2, M-CSF, and GM-CSF (Fig. 2b). M-CSF, GM-CSF were likely produced from the KPrLG cells themselves as heightened levels of both these chemokines could be detected in cultured supernatant after p53 restoration in vitro (Fig. 2c). Regardless of origin, the biological activity of these chemoattractants, the rapid infiltration of CD45-positive leukocytes into lung adenocarcinomas after p53 reactivation, and the subsequent fibroblast recruitment during tumor regression suggests that p53 orchestrates an immune reaction in the lung microenvironment that leads to the surveillance and destruction of cancer cells by the innate immune system followed by a fibrotic healing process.

### A complex pulmonary immune reaction ensues after p53 reactivation

**Fig. 3:**
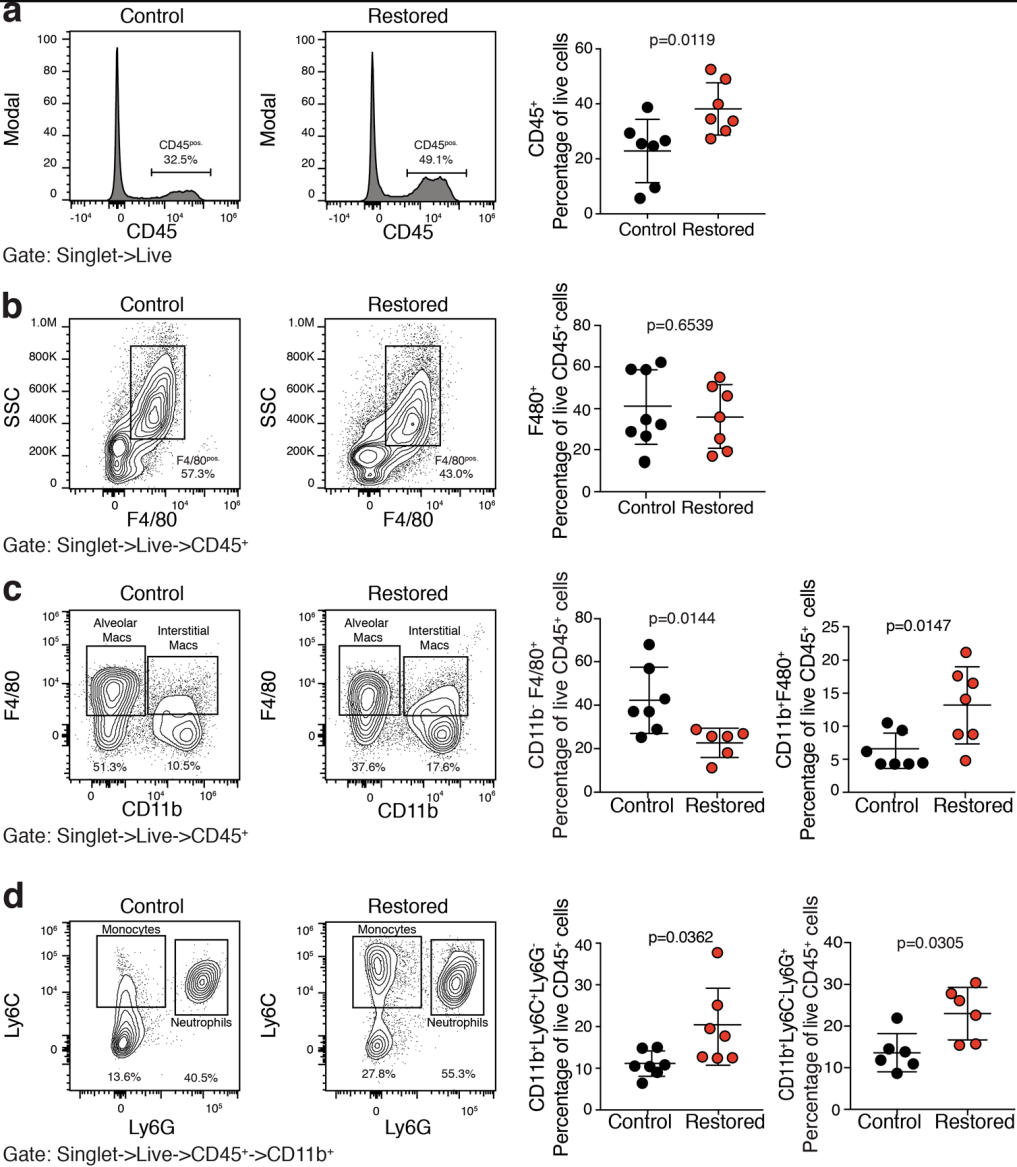
Restoration of p53 in lung adenocarcinomas leads to potent immune inflammatory response in the lung. Cell suspensions from *Rag1*^*-/-*^ mouse lungs bearing orthotopically transplanted KPr adenocarcinomas were treated with corn oil (Control) or tamoxifen (Restored) and subjected to multiparameter flow cytometry. **a** Cells gated to include live singlets are plotted on histograms for CD45 and quantified at right. **b** CD45^pos.^ cells from **a** plotted on F4/80 X SSC. F4/80^pos.^ cells gated and quantified at right. **c** CD45^pos.^ cells from **a** plotted on CD11b X F4/80. CD11b^neg.^;F4/80^pos.^ alveolar macrophages at bottom left and CD11b^pos.^F4/80^pos.^ CD11b^pos.^ macrophages gated and quantified at bottom right. **d** CD11b^pos.^ cells from **a** plotted on Ly6G X Ly6C. CD11b^pos.^Ly6G^pos.^ neutrophils ^and^ CD11b^pos.^Ly6C^pos.^ monocytes cells are gated and quantified at right. Analysis of significance between control and restored groups was performed by *t*-test

We next wanted to determine the effect of p53 reactivation in KPrLG cells on the frequency of specific innate immune cell populations in the lung microenvironment. *Rag1*^*-/-*^ mice orthotopically transplanted with KPrLG cells were treated as above with vehicle or tamoxifen and then lungs were harvested for analysis 6 days after p53 reactivation. We performed multiparameter flow cytometry on single cell suspensions of lung tissue. Consistent with the histological analysis described above, we detected a significantly increased number of CD45-positive cells after p53 reactivation (Fig. 3a). However, the increased frequency of CD45-positive cells did not represent a single cell type but instead a complex mixture of multiple innate immune cells. These cells included CD11b^pos.^;Ly6G^pos.^ neutrophils, CD11b^pos.^;Ly6C^pos.^ monocytes, and CD11b^pos.^;F4/80^pos.^ macrophages (Fig. 3b–d). Interestingly, the overall number of F4/80-positive cells in the lung was not significantly changed due to a significant decrease in the frequency of CD11b^neg.^;F4/80^pos.^ alveolar macrophages (Fig. 3b, c). These data suggest that p53 reactivation in KPrLG cells has a widespread and complex effect on the constituents of innate immune cell types in the lung milieu.

### B220-positive NK cell subsets emerge after p53 restoration

**Fig. 4:**
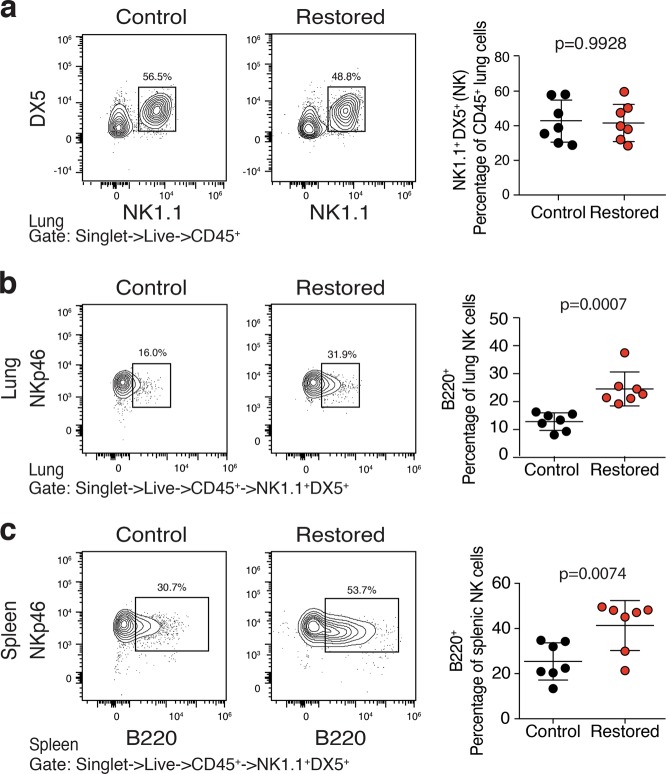
NK cells express B220 activation mark after p53 restoration. Cell suspensions from *Rag1*^*-/-*^ mouse lungs bearing orthotopically transplanted KPr lung adenocarcinomas treated with corn oil (Control) or tamoxifen (Restored) were subjected to multiparameter flow cytometry. **a** Cells gated to include live singlets that are CD45^pos.^ are plotted on NK1.1 x DX5. NK1.1^pos.^;DX5^pos.^ cells are gated (top) and then plotted for B220^pos.^;NKp46^pos.^ histogram. **b** Splenocytes also shown for comparison. **c** Analysis of significance between control and restored groups was performed by *t*-test

To date, immune-mediated tumor clearance following p53-induced senescence has centered on the recruitment and activation of NK cells and their subsequent killing of senescent tumor cells^11–13^. However, surprisingly, based on expression of NK1.1, DX5, or NKp46, we did not observe a significantly altered frequency of NK cells after p53 reactivation (Fig. 4a and not shown). Therefore, we set out to determine whether NK cells had an altered cell surface phenotype associated with their increased activity. Although typically used as a B cell marker, when expressed on NK cells, B220 marks cells with increased tumoricidal activity, ability to produce large amounts of interferon γ, and the controversial potential to present antigen via MHC-I^17–21^. Although B220 expression was largely absent on NK1.1^pos.^ cells prior to p53 reactivation, we found that a significant fraction of NK cells in the lung expressed B220 on their surface after p53 reactivation (Fig. 4b). Interestingly, we also observed an even more significant increase in B220 expression on splenic NK cells after p53 restoration suggesting a systemic response to p53 reactivation (Fig. 4c). These data suggest that although NK cells may not appreciably traffic to or expand within tumor sites and lung tissue after p53 reactivation, they alter their surface phenotype indicating they may instruct or carry out tumor cell destruction.

**Fig. 5:**
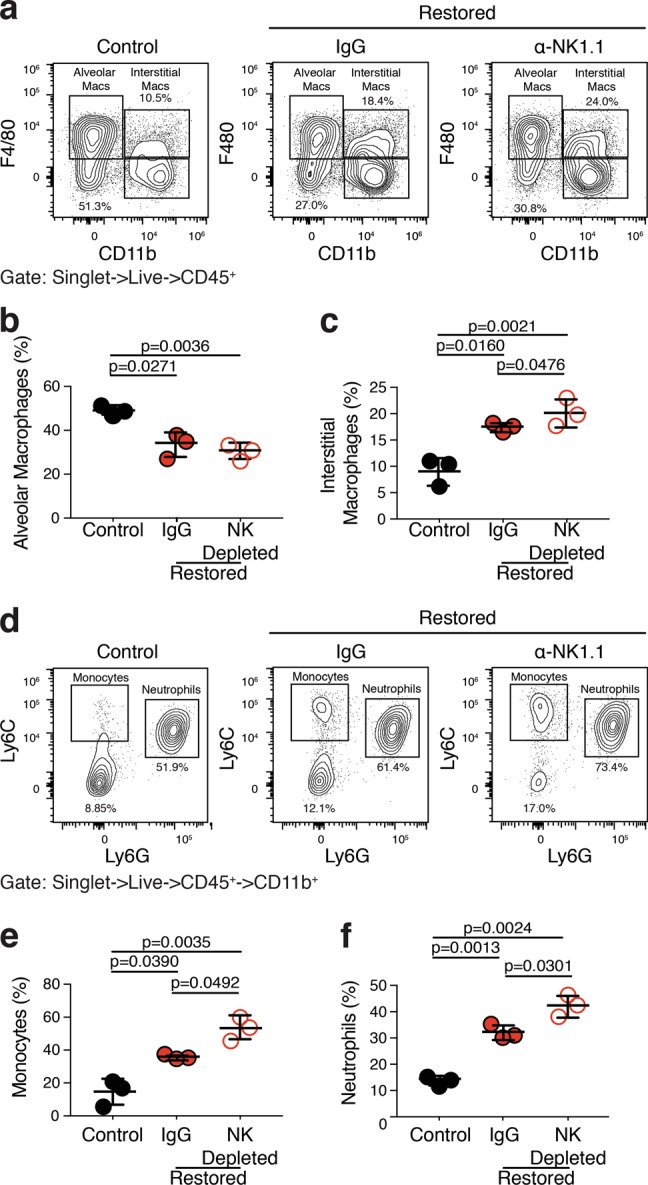
NK cells limit a p53-mediated immunoinflammatory reaction that is associated with lung adenocarcinoma regression. Cell suspensions from the lungs of KPr tumor-bearing *Rag1*^*-/-*^ mice treated with corn oil (Control), tamoxifen (Restored), and tamoxifen, and anti-NK1.1 (Restored NK Depleted) were subjected to multiparameter flow cytometry. **a** Cells gated to include live singlets that are CD45^pos.^ are plotted on CD11b X F4/80. **b** Alveolar macrophages (CD11b^neg.^; F4/80^pos.^) are quantified from **a**. **c** Interstitial macrophages (CD11b^pos.^;F4/80^pos.^) are quantified from **a**. **d** CD11b^pos.^ cells from **a** are plotted on Ly6G X Ly6C. **e** Monocytes (CD11b^pos.^; Ly6C^pos.^; Ly6G^neg.^) are quantified from **d**. **f** Neutrophils (CD11b^pos.^; Ly6C^pos.^; Ly6G^pos.^) are quantified from **d**. Analysis of significance between control and restored treatment groups was performed by *t*-test

To begin to assess the impact that NK cells have on the broader immune response during p53-mediated lung tumor regression, we transplanted cohorts of C57BL/6: *Rag1*^*-/-*^ mice with KPrLG cells as before and treated each cohort with either isotype control or anti-NK1.1 NK cell depletion antibody. Intraperitoneal delivery of a NK1.1 antibody efficiently depleted NK cells in the lung and spleen over the 10 day time course of the experiment (Fig. S2). After 2 days, subsets of animals were treated with vehicle control or tamoxifen to reactivate p53. Two and 6 days later we harvested lung tissue from each mouse and made single cell suspensions to profile immune cells by flow cytometry. Similar to that shown in Fig. 3, we observed a significant increase in the frequency of neutrophils (CD11b^pos.^; Ly6G^pos.^; Ly6C^pos.^), monocytes (CD11b^pos.^; Ly6G^neg.^; Ly6C^pos.^), and CD11b^pos.^ macrophages (CD11b^pos.^ F4/80^pos.^) but a loss of alveolar macrophages (CD11b^neg.^; F4/80^pos.^) after p53 reactivation (Fig. 5a–f and Fig. S3). Interestingly, depletion of NK cells with NK1.1 antibody significantly augmented the gains in neutrophils, monocytes, and CD11b^pos.^ macrophages (Fig. 5c, e, f). These data suggest that NK cells respond within the lung tissue microenvironment during immune-mediated tumor cell clearance but paradoxically act to dampen the pro-inflammatory innate immune response that ensues after p53 restoration in lung adenocarcinoma cells.

### p53 reactivation induces rapid and efficient tumor cell clearance in the absence of NK cells

**Fig. 6:**
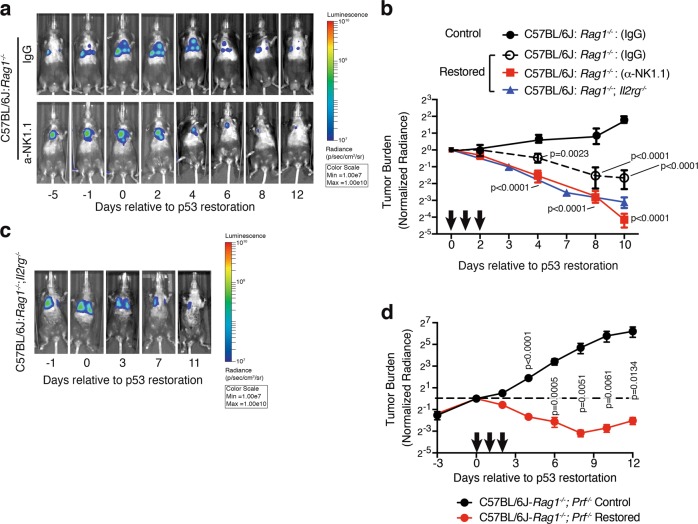
NK cells limit p53-mediated lung adenocarcinoma regression. Cohorts of mice were depleted for NK cells with α-NK1.1 antibody, or treated with an isotype control IgG and then orthotopically transplanted with KPr adenocarcinoma cells. Mice were treated with corn oil or tamoxifen and tumor burden was observed by bioluminescent imaging. **a** Representative bioluminescent images of C57BL6/J:*Rag1*^*-/-*^ mice transplanted with KPrLG cells. *Rag1*^*-/-*^ mice were inoculated with IgG or α-NK1.1 NK cell-depleting antibody on days −2, 2, and 5 (**a**, **b**). The initial treatment with corn oil (Control) or tamoxifen (Restored) is defined as day 0. Subsequent treatments indicated by arrows on *X*-axis. Images acquired relative to day 0 are from the same animal at the indicated time points. **b** Quantification of tumor burden from **a**. **c** Representative bioluminescent images of C57BL6/J:*Rag1*^*-/-*^;*Il2rg*^*-/-*^ mice transplanted with KPrLG cells. Quantification of tumor burden is overlaid for comparison in **b**. Analysis of significance between control and restored groups was performed by two-way ANOVA. **d** Quantification of tumor burden from C57BL/6: *Rag1*^*-/-*^*; Prf*^*-/-*^ transplanted with KPrLG cells and treated with corn oil (Control) or tamoxifen (Restored) at time points indicated by arrows. Values are normalized to day 0 measurements. Analysis of significance between control and restored groups was performed by *t*-test

To determine the role that NK cells play during p53-mediated regression of lung adenocarcinoma, we depleted NK cells from tumor-bearing *Rag1*^*-/-*^ mice prior to p53 reactivation as above. As expected, reactivation of p53 in KPrLG cells led to the gradual regression of tumors in mice given an isotype control antibody. However, in C57BL/6: *Rag1*^*-/-*^ mice that were depleted of NK cells with NK1.1 antibody, we surprisingly observed a more rapid and thorough decline in luminescent signal after p53 reactivation (Fig. 6a, b). To more stringently assess the impact of NK cell loss on regression of lung adenocarcinoma cells following p53 reactivation, we also transplanted KPrLG cells into C57BL/6: *Rag1*^*-/-*^*; Il2rg*^*-/-*^ mice that additionally lack the common gamma chain (γ_c_) that is necessary for NK cell development and signaling through multiple cytokine receptors. C57BL/6: *Rag1*^*-/-*^*; Il2rg*^*-/-*^ therefore lack B, T, and NK cells. Reactivation of p53 in KPrLG-bearing C57BL/6: *Rag1*^*-/-*^*; Il2rg*^*-/-*^ mice also resulted in rapid tumor regression (Fig. 6b, c). Indeed, the rate and degree of tumor regression after p53 reactivation was indistinguishable from that observed in C57BL/6: *Rag1*^*-/-*^ mice that were depleted of NK cells with NK1.1 antibody (Fig. 6b). These observations were counter to our expectation and not only demonstrate that NK cells are not required for tumor regression after p53 reactivation in lung adenocarcinoma cells, but also suggest that they actively limit tumor cell regression. We additionally explored the effect that strain background might have on the effects of NK cell loss in the regression of lung adenocarcinoma after p53 reactivation. We transplanted KPrLG cells into mice that are of the NOD background and had *Rag1*^*-/-*^*; Il2rg*^*-/-*^ (NRG) or *Pkrd*^*scid/scid*^*; Il2rg*^*-/-*^ (NSG) genotype. Again, p53 reactivation led to significant regression of KPrLG tumor cells in both these mouse backgrounds (Fig. S4a, b). Similar to tumors receding in C57BL/6: *Rag1*^*-/-*^ mice, tumor regression in C57BL/6: *Rag1*^*-/-*^*; Il2rg*^*-/-*^ mice also was typified by a significant culling of GFP-positive tumor cells and a concomitant increase in fibrosis (Fig. S4c). Consistent with the increased frequency of inflammatory cells in *Rag1*^*-/-*^ mice lacking NK cells, we detected significant enrichment for GM-CSF, CCL2, CXCL1, and G-CSF that that are likely responsible for their attraction in lungs of C57BL/6: *Rag1*^*-/-*^*; Il2rg*^*-/-*^ mice after p53 restoration (Fig. S4d). Finally, because granule mediated cell killing is critical for destruction of senescent liver cancer cells^12^, we assessed the impact of perforin deficiency on tumor regression by generating C57BL/6: *Rag1*^*-/-*^*; Prf*^*-/-*^ mice and transplanting them with KPrLG lung adenocarcinoma cells. Reactivation of p53 in KPrLG cells that were transplanted into C57BL/6: *Rag1*^*-/-*^*; Prf*^*-/-*^ mice resulted in rapid and robust tumor cell clearance similar to NK cell deficient animals (Fig. 6d). These data strongly demonstrate that NK cells are not required for lung adenocarcinoma regression in the context of the lung and support a model where NK cell-dependent cytotoxicity acts on the tumor microenvironment to dampen the rate and extent of tumor cell clearance after p53 reactivation.

### NK cells limit cancer cell engraftment in the lung

**Fig. 7:**
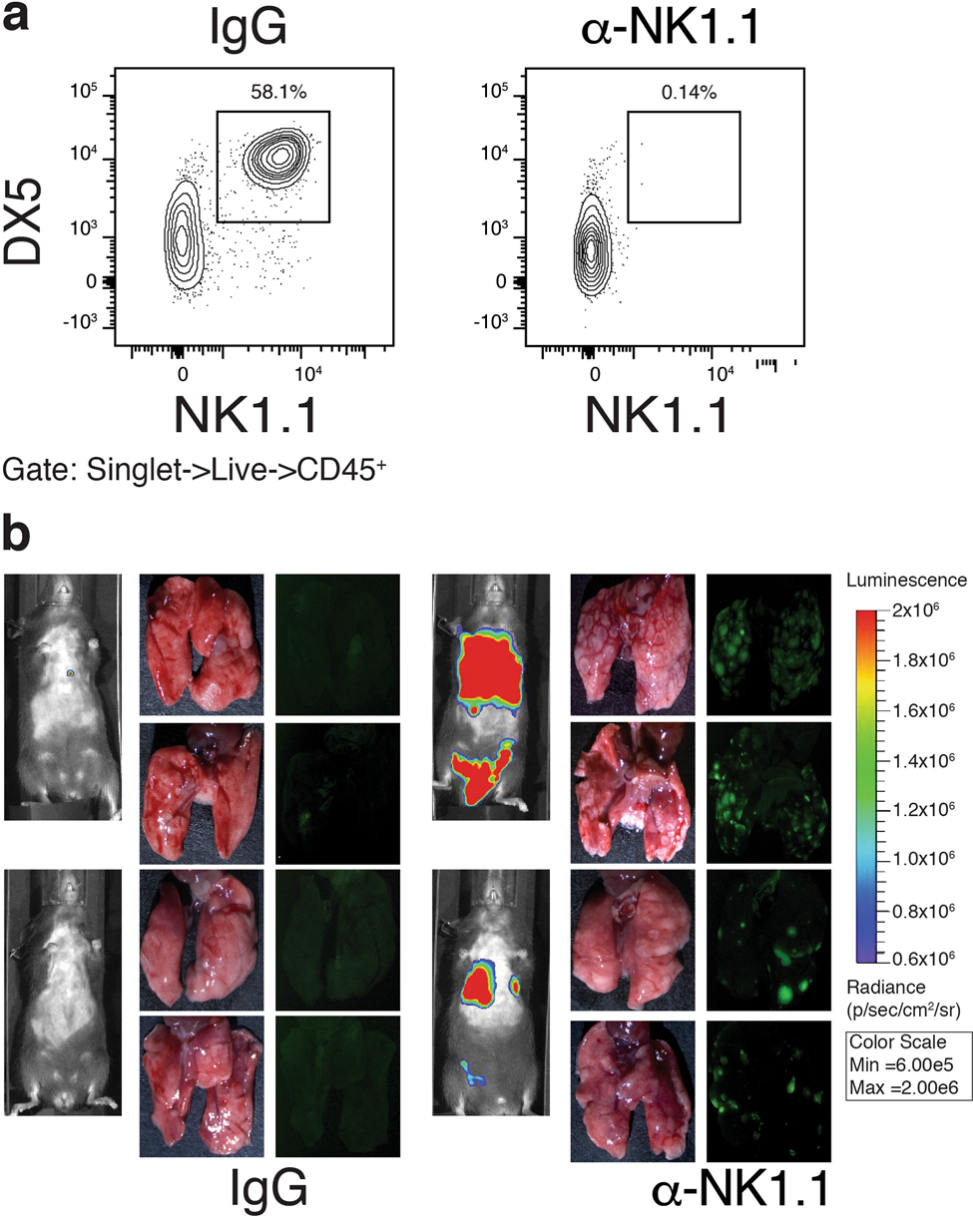
NK cells limit tumor cell seeding in the lung. **a** Detection of DX5^pos.^;NK1.1^pos.^ NK cells in C57BL6/J:*Rag1*^*-/-*^ mice inoculated with IgG or anti-NK1.1 NK cell-depleting antibody. Fluorescent stereomicroscopic imaging of mouse lungs seeded with KPrLG tumor cells. **b** *Rag1*^*-/-*^ mice were pretreated with control IgG (**b**) or to NK1.1 cell depletion antibody (**c**). Two representative mice are shown from *n* = 3 each. Images are from 13 days following KPrLG cell injection and filtered for GFP and luminescent imaging

During the course of these orthotopic transplantation experiments, we noted that transplantation of KPrLG cells into *Rag1*^*-/-*^*; Il2rg*^*-/-*^ mice was more efficient than transplantation into *Rag1*^*-/-*^ mice regardless of the strain background (Table 1). On average, we found that *Rag1*^*-/-*^ mice developed well-established tumors 24.8 days following intravenous tail vein injection. In contrast, all strains that lack NK cells due to *Il2rg*^*-/-*^ genotype or critical NK cell functions (*Prf*^*-/-*^) developed well-established tumors approximately 12 days post-transfer (*p* < 0.0001). More rapid engraftment suggests that NK cells can in fact surveil KPrLG cells in the lung, but that they do so during, or shortly after the event of their seeding and in a p53-independent manner. To test this hypothesis more directly, we administered anti-NK1.1 NK cell depletion antibody once every three days starting 3 days prior to tumor cell injection and observed mice for tumor development in the absence of NK cells (Table 1). As in *Il2rg*^*-/-*^ mice that are genetically deficient for NK cell production, tumor cells engrafted more efficiently in *Rag1*^*-/-*^ mice that had been depleted of NK cells with NK1.1 antibody compared to control antibody treated Rag^-/-^ mice (Fig. 7b,c). These observations are consistent with a model where NK cells have the capacity to sense newly invading cancer cells in the lung tissue. However, once established, proliferating KPrLG lung adenocarcinoma cells are not recognized by NK cells for destruction prior to or following p53 activation.

**Table 1:** NK cells limit initial cell engraphment into the lungs

Strain	Genotype	Average (SD) days to engraphment^a^	Number of experiments	Number of mice	*p* value (ANOVA) vs. C57BL6/J: *Rag1*^*-/-*^
C57BL6/J	*Rag1* ^*-/-*^	24.8 (5.3)	22	266	NA
C57BL6/J	*Rag1* ^*-/-*^ *;Il2rg* ^*-/-*^	12.1 (4.1)	7	50	<0.0001
C57BL6/J	*Rag1* ^*-/-*^ *;Prf* ^*-/-*^	10 (0)	1	12	<0.05
NOD	Prkd^scid/scid^;Il2rg_-/-_	8 (0)	1	9	<0.01
NOD	*Rag1* ^*-/-*^ *;Il2rg* ^*-/-*^	12 (1.8)	7	106	<0.0001

^a^Engraphment defined as luminescence radiance (p/sec/cm^2^/sr) greater than 10^8^

## Discussion

The concept of immune surveillance of senescent cells has emerged as a mechanism where senescent cells communicate with their microenvironment to recruit immune cell types that ultimately remove, or cull them from their tissue residence^22^. Chronic exposure to liver damaging toxins induces fibrosis that is kept in check by activation of a senescence program in hepatic stellate cells. These senescent, matrix-depositing cells secrete inflammatory molecules that recruit a multitude of innate immune cell types; however, their clearance was strictly dependent on the presence of NK cells^23^. In cancer, pre-malignant oncogene expressing cells or established cancers engineered to reintroduce p53 activity in a temporally controlled manner each induced a p53-controlled senescence program that ultimately leads to immune-mediated clearance^11,13,24^. In senescent pre-malignant hepatocytes, immune clearance required both antigen-specific CD4 T cells for recognition and to provide stimulation to recruited monocytes and macrophages that implemented the cell killing functions^24^. In hepatocellular carcinomas, reintroduction of p53 activity induced a senescence response that recruited multiple innate immune cell types to eliminate tumors from these mice that was dependent on the expression of NKG2D receptors on NK cells and the secretion of CCL2 from senescent tumor cells^11,13^. Our work in lung adenocarcinoma here contrasts these observations. Although we showed that p53 reactivation in lung adenocarcinoma cells prompted a rapid inflammatory immune response similar to that observed in the liver, we found that NK cells did not play an active role in the culling of these tumor cells from the lung. Instead, we found that the lack of NK cells or perforin-mediated cytolysis promoted a more effective clearance of lung adenocarcinoma cells from the lung that was likely due to a heightened immunoinflammatory reaction subsequent to p53 activation. Our data therefore suggest that NK cells act to limit immunoinflammation and tumor regression in this context. Recent studies in Kras-driven and p53-deficient lung adenocarcinomas demonstrated that treatment with a combination of inhibitors that target MEK1/2 of the mitogen-activated protein kinase pathway and cyclin-dependent kinase (CDK) 4 and CDK6 could induce an RB-dependent senescence program that would activate an NK-cell-dependent destruction of tumors cells^25^. Taken together with our data here, it is interesting to speculate the existence of distinct senescence-associated immune-surveillance programs within similar lung adenocarcinoma cells that are controlled by RB or p53.

Therapies that target the patient’s immune system and inspire tumoricidal activities offer a potentially more widespread approach to lung adenocarcinoma treatment^26^. Current immunotherapies focus on relieving immune checkpoint blockades that restrict the tumoricidal activity of adaptive immune cell types, namely T cells. Despite clear benefits, for poorly understood reasons, targeting immune checkpoints is only effective in a subset of patients and often lacks durable responses^26^. However, therapeutic modulation of the innate immune system offers an orthogonal approach that can be important for establishing more widespread or durable effects^27^. Although our study has focused entirely on in vivo contexts where the adaptive immune system is ablated, the data suggest the intriguing potential that mimicking the effects of p53 reactivation in lung adenocarcinoma cells and/or limiting the NK-cell-dependent dampening of the immune inflammatory microenvironment. These innate immune cell-focused interventions may work in concert with those that target the adaptive arm of the immune system to yield better outcomes for patients.

## Methods

### Animals

Animal studies were approved by the Committee for Animal Care and conducted in compliance with the Animal Welfare Act Regulations and other federal statutes relating to animals and experiments involving animals and adheres to the principles set forth in the Guide for the Care and Use of Laboratory Animals, National Research Council, 1996. C57BL/6:*Rag1*^*tm1Mom*^ were obtained from the Jackson Laboratory (Bar Harbor, ME, USA) and maintained in-house. B6.129S4:*Il2rg*^*tm1Wjl*^/J and C57BL/6:*Prf1*^*tm1Sdz*^/J mice were obtained from Jackson Laboratory and crossed to C57BL/6-*Rag1*^*tm1Mom*^ mice to generate C57BL/6:*Rag1*^*tm1Mom*^; *Il2rg*^*tm1Wjl*^ and C57BL/6:*Rag1*^*tm1Mom*^; *Prf1*^*tm1Sdz*^/J mice. NOD.Cg:*Rag1*^*tm1Mom*^; *Il2rg*^*tm1Wjl*^/SzJ and NOD.Cg:*Prkdc*^*scid*^*; Il2rg*^*tm1Wjl*^/SzJ were obtained from the Jackson Laboratory. Tamoxifen (Sigma, St. Louis, MO, USA) was administered once tumors reached a radiance value greater than 10^8^ p/sec/cm^2^/sr except for nude mice which started at 10^7^ p/sec/cm^2^/sr. Mice were grouped randomly after sample size estimation. Mice were grouped to include an equal distribution sex and age across treatment groups. Male and female mice between the ages of 6 and 12 weeks were used for experiments. Tamoxifen was administered by oral gavage at a concentration of 20 mg/mL in a 200 μl final volume. To deplete NK cells, anti-NK1.1 (PK136) antibody or mouse IgG2a control (BioXcell, Lebanon, NH, USA) was administered intraperitoneally to mice at indicated time points at a concentration of 300 µg/ml.

### Cells

KPr and KPrLG cell lines were derived from *Kras*^*LA2/+*^*;Trp53*^*LSL/LSL*^*;Rosa26*^*CreERT2/CreERT2*^ adenocarcinomas^14^. KPrLG cells were generated by retroviral transduction of MSCV-based particles containing a MSCV-driven luciferase-IRES-GFP construct (Addgene, Watertown, MA, USA). Two independent cell lines (KPr8LG and KPr10LG) were both used routinely in cell transplant studies with indistinguishable results. All cell lines were grown in DMEM culture media with 10% FBS and 10 mg/ml gentamicin. For the orthotopic tumor model, cells were harvested by trypsinization, washed twice with PBS, and adjusted to a concentration of 2 × 10^6^ cells/ml. 4 × 10^5^ cells in 200 μl PBS were intravenously injected into each mouse via the tail vein. For cell death assays, cell lines were harvested and stained as detailed in the flow cytometry methods section. Cells were stained with SAβ-galactosidase to evaluate cellular senescence. Briefly, cells were washed and incubated with β-galactosidase staining solution (Cell Signaling, Danvers, MA, USA). Following staining, cells were washed and fixed in 4% PFA for 5 min. Samples were then washed with PBS and imaged on a Leica DMI6000B inverted light microscope.

### Optical bioluminescence imaging and fluorescent imaging

Whole body imaging was performed to detect luciferase reporter activity using the Xenogen IVIS® in vivo Imaging System (Caliper Life Science, Waltham, MA, USA). Mice were intraperitoneally injected with 200 μl D-luciferin potassium salt dissolved in PBS at a concentration of 30 mg/ml (Gold Bio, St. Louis, MO, USA). Mice were anesthetized with isoflurane (Henry Schein, Melville, NY, USA) at 2.5% mixed with oxygen in a separate induction chamber. Animals were moved to the imaging chamber and provided 1.0% maintenance anesthesia within the imaging chamber. Animals were imaged 10 min after D-luciferin injection. Pseudocolored bioluminescence images were obtained using Living Image (PerkinElmer, Waltham, MA, USA) software. Measurements of total flux were obtained using Living Image software. Measurements are normalized to flux values obtained on day 0 for each mouse. For GFP^pos.^ lung images, lungs were removed from mice and imaged on a Leica (Buffalo Grove, IL, USA) M80 stereo microscope.

### Histology and immunohistochemistry

Lung tissues were inflated through the trachea with 4% neutral buffered formalin and soaked overnight at 4 °C. Tissue were changed to 70% ethanol for storage until paraffin embedding. Tissues were sectioned into 4-µm-thick sections, deparaffinized in xylene, and rehydrated in graded ethanol prior to staining. H&E and trichome stains were performed by University of Pennsylvania Cancer Biology Histology core. For all cell marker specific antibody stains, sections were subjected to antigen retrieval steam heating in Buffer A antigen retrieval solution (Electron Microscopy Sciences, Hatfield, PA, USA) or incubation in 10 µg/ml proteinase K in TE 8 buffer at 37 ℃. After blocking for 20 min at room temperature in dual endogenous enzyme block (Dako, Santa Clara, CA), slides were blocked for 20 min each with avidin and biotin blocks (Vector Labs, Burlingame, CA, USA) and for 30 min with serum-free protein block (Dako). Slides were incubated overnight at 4 °C with primary antibodies against CD45 (Biolegend, San Diego CA, USA; 30-F11; 103102; 1:50; 10 µg/ml pK retrieval 10 min), GFP (abcam, Cambridge, MA, USA; ab13970; 1:1000; heat citrate retrieval), or smooth muscle actin (SMA) (abcam; ab5694; 1:200; heat citrate retrieval). Slides were then incubated at room temperature for 1 h with anti–rabbit (VECTASTAIN ABC Rabbit IgG detection kit; Vector Laboratories), anti-rat secondary antibodies (VECTASTAIN ABC Rabbit IgG detection kit; Vector Laboratories), or anti-chicken (Jackson; 1:1000). Staining was detected using Immpact DAB substrate (Vector) and counterstained with hematoxylin. For CD45, slides were stained using the Mouse on Mouse (MOM) Kit (Vector) and ABC Elite (Vector) according to manufacturer’s instructions. Photomicrographs were captured on a Leica DMI6000B inverted light microscope.

### Cellular harvest and flow cytometry

Lungs were harvested at indicated time points and digested with buffer containing 10 mg/ml collagenase type 4 (Worthington Chemicals, Lakewood, NJ, USA) and 2 mg/ml DNase (Sigma) using the gentleMACS™ Octo Dissociator (Miltenyi Biotec, Auburn, CA, USA). Specimens were passed through a 100-μM cell strainer and centrifuged at 1000 rpm for 5 min. Samples were counted using a Z-Series Coulter Counter (Beckman Coulter, Brea, CA, USA). Equal cell numbers of each sample were stained for flow cytometry analysis. Cells were resuspended in buffer contained 0.1% serum and sodium azide. After blocking FcγRIII/II with an anti-CD16/CD32 mAb (eBioscience, San Diego, CA; 1:500), cells were labeled with the following antibodies: CD45 FITC (Biolegend; 30-F11; 1:200), CD49b FITC (Biolegend; DX5; 1:200), F4/80 FITC (Biolegend; BM8; 1:100), Ly6G FITC (Biolegend; 1A8; 1:200), B220 Pe-Cy5 (BD Pharm; RA3-682; 1:200), CD11b Percp-Cy5.5 (Biolegend; M1/70; 1:200), Ly6G PE (1A8, Invitrogen, Carlsbad, CA, USA; 1:200), NK1.1 PE (Biolegend; PK136; 1:200), Ly6C PE-Cy7 (Biolegened; HK1.4; 1:300), NK1.1 PE-Cy7 (eBioscience; PK136; 1:200), 7AAD (BD Pharm, San Jose, CA, USA; 1:100), CD45 APC (Biolegend; 30-F11; 1:200), CD49a APC (Biolegend; HMα1; 1:200), CD49b APC (Biolegend; DX5; 1:200), F4/80 APC (Biolegend; BM8; 1:100), CD11b Super Bright 702 (Invitrogen; M1/70; 1:200), CD45 APC-efluor780 (Invitrogen; 30-F11; 1:200), Zombie NIR (Biolegend; 1:200), NKp46 eFluor450 (ebioscience; 29A1.4; 1:200), CD11b Super Bright 500 (Invitrogen; M1/70; 1:200), and CD45 Super Bright 645 (Invitrogen; 30-F11; 1:200). BrdU APC staining was carried out according to manufacture’s instructions (BD Pharm). Flow cytometry was performed on the Attune NxT Flow Cytometer (ThermoFisher Scientific, Waltham, MA, USA). Data were analyzed using FlowJo (Version 10.5.2 TreeStar, Ashland, OR, USA).

### Multiplex cytokine assay

Conditioned cell culture supernatant from KPr cells or bronchioalveolar lavage (BAL) fluid or lung tissue lysates from tumor-bearing *Rag1*^*-/-*^ mice for multiparameter cytokine and chemokine detection assay after p53 reactivation. For lung tissue lysates, lungs were homogenized using a motorized tissue grinder (Fisherbrand, Pittsburgh, PA, USA) and resuspended in buffer containing Tris HCl, Tween 20, and NaCl. Samples were all diluted to an equal concentration for analysis. Multiplexing LASER Bead Assay was performed by Eve Technologies (Calgary, AB Canada).

### Statistical analysis

All experiments were conducted a minimum of three times. The results were calculated as mean ± standard deviation. An unpaired Student’s *t*-test was performed when variance was not significant and a Welch’s *t*-test was performed for experiments with significance differences in variance. Two-way ANOVA were used for statistical analysis as indicated in figure legends for experiments with three or more groups. Statistical analysis was performed using Prism (Graphpad Software, San Diego, CA).

## Supplementary information

Supplemental Figure Legends

Supplemental Figure 1

Supplemental Figure 2

Supplemental Figure 3

Supplemental Figure 4
