# Supplementary material for: Natural killer cells limit the clearance of senescent lung adenocarcinoma cells
Source: Oncogenesis. 2019 Apr 1;8(4):24. doi: 10.1038/s41389-019-0133-3 (PMC6443683; doi:10.1038/s41389-019-0133-3)
Supplement: Supplementary file 1 — Supplemental Figure Legends [file 41389_2019_133_MOESM1_ESM.docx]

**Supplementary Fig. 1:** Restoration of p53 induces cell cycle arrest and senescence with minimal evidence of cell death in KPr lung adenocarcinoma-derived cell lines. **a)** Cell proliferation/cycle analysis in two independently derived KPr cell lines (KPr8 and KPr10). Flow cytometry plots indicating DNA content (7-AAD) and BrdU incorporation cultures 72 hours after treatments with ethanol (Control) or 4-OHT (Restored) to reactivate p53. **b)** Quantification of BrdU positive cells gated in (a) with significance determined by unpaired t test. Data is normalized to control samples. **c)** Unfixed, parallel cultures from (a) were assessed for cell death using a viability dye (7-AAD) exclusion method. Viable fraction relative to control is plotted with significance determined by unpaired t test. Data is normalized to control samples. **d)** Senescence associated β-galactosidase assay on KPr8 and KPr10 cells 10 days after p53 restoration.

**Supplementary Fig. 2:** Detection of DX5^pos.^;NK1.1^pos.^ NK cells C57BL6/J:*Rag1^-/-^* mice inoculated with IgG or anti-NK1.1 NK cell-depleting antibody, transplanted with KPrLG cells, and treated with corn oil (Control) or tamoxifen (Restored). Representative flow plots from lung cells (b) and spleen cells (c) in control or anti-NK1.1 treated mice. Percentages of lung NK cells (d) and spleen NK cells (e) from control and depleted are summarized.

**Supplementary Fig. 3:** As in Fig. 4 but at two days post-p53 restoration. Cell suspensions from *Rag1^-/-^* mouse lungs bearing orthotopically transplanted KPr adenocarcinoma cells treated with corn oil (Control) or tamoxifen (Restored) were subjected to multi-parameter flow cytometry two days after p53 reactivation. Cohorts of mice were depleted for NK cells with α-NK1.1 antibody, isotype control IgG, or untreated (Control). **a)** Cells gated to include live singlets that are CD45^pos.^ are plotted on CD11b X F4/80. Alveolar macrophages are gated and quantified in **(b)**. CD11b^pos.^;F4/80^pos.^ CD11b^pos.^ macrophages are gated and quantified in **(c)**. **d)** CD11b^pos.^ cells from **(a)** are plotted on Ly6G X Ly6C. CD11b^pos.^; Ly6C^pos.^; Ly6G^neg.^ monocytes are quantified and plotted in **(e)**. CD11b^pos.^; Ly6C^pos.^; Ly6G^pos.^ neutrophils are quantified and plotted in **(f)**. Analysis of significance between genotypes and antibody treatment groups was performed by t test.

**Supplementary Fig. 4:** Quantification of bioluminescent images from *Pkrd^scid/scid^; Il2rg^-/-^* (NSG) **(a)** and NOD:*Rag1^-/-^; Il2rg^-/-^* (NRG) **(b)**. Corn oil or tamoxifen was administered on day 0. Subsequent treatments indicated by arrows on x-axis. Analysis of significance between control and restored groups was performed by two-way ANOVA. **c)** Histological sections from Control and p53 Restored tumors at Day 5, and Day 10. H&E, IHC for GFP, and Trichrome staining for collagen deposition is shown. (d) Multiparameter cytokine detection in lung lysates of Control and tamoxifen treated mice on days 2, 5, and 10 post-p53 restoration. Significantly changing cytokines are indicated (GM-CSF, CCL2, CXCL1, and G-CSF).
