# Supplementary figures and images for: Natural killer cells limit the clearance of senescent lung adenocarcinoma cells
Source: Oncogenesis. 2019 Apr 1;8(4):24. doi: 10.1038/s41389-019-0133-3 (PMC6443683; doi:10.1038/s41389-019-0133-3)

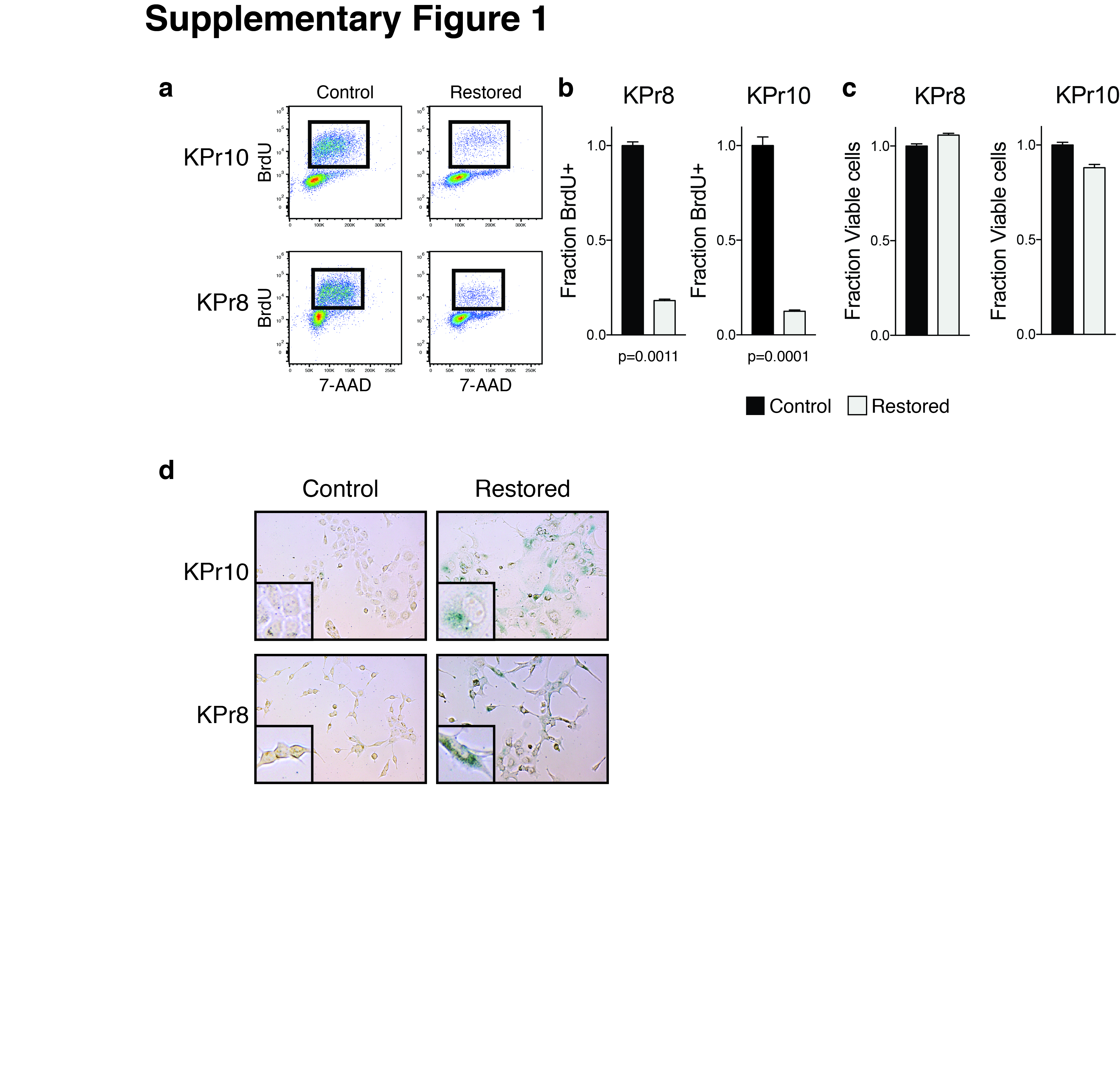

Supplement: Supplementary file 2 — Supplemental Figure 1 [file 41389_2019_133_MOESM2_ESM.jpg]

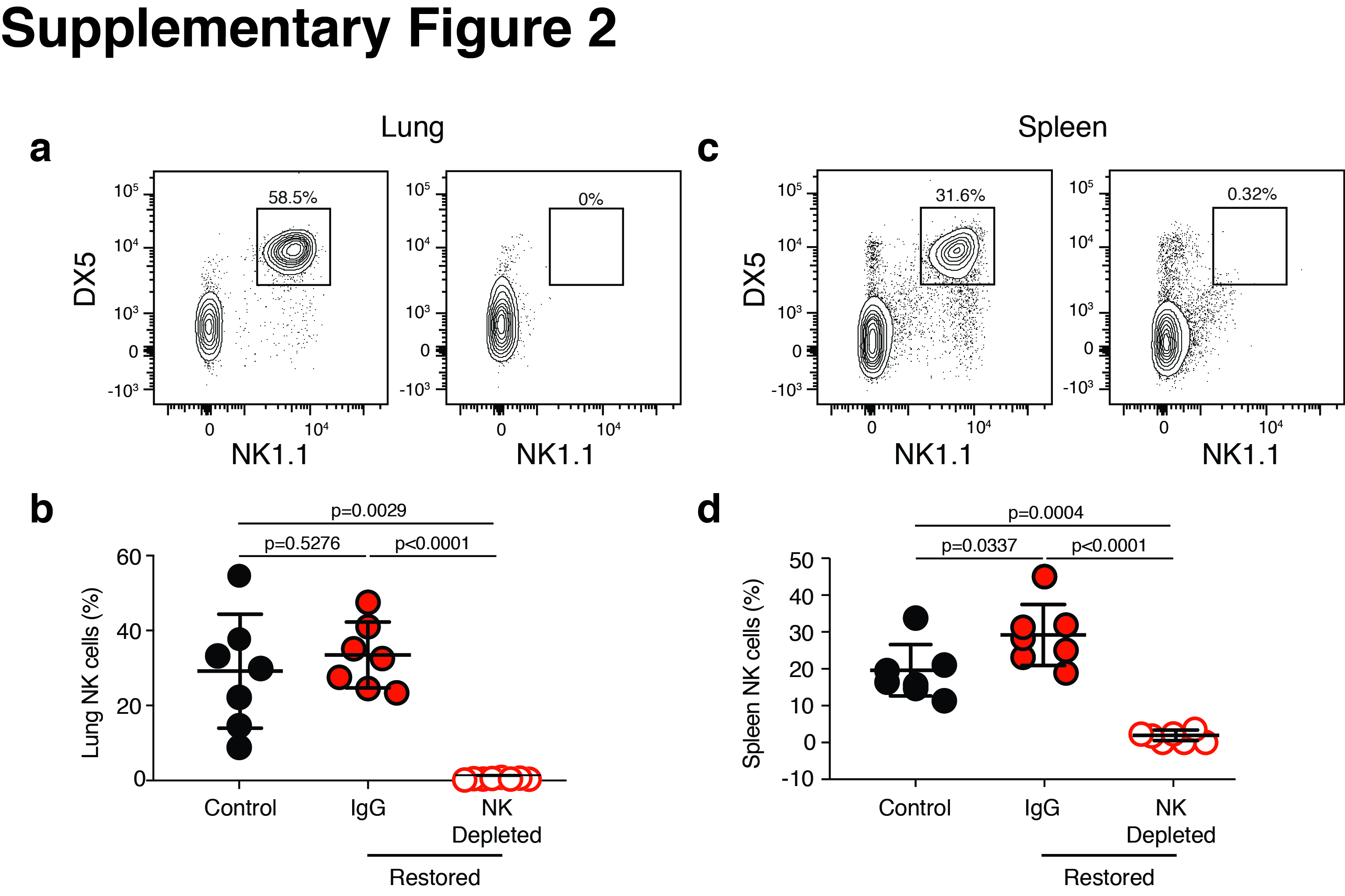

Supplement: Supplementary file 3 — Supplemental Figure 2 [file 41389_2019_133_MOESM3_ESM.jpg]

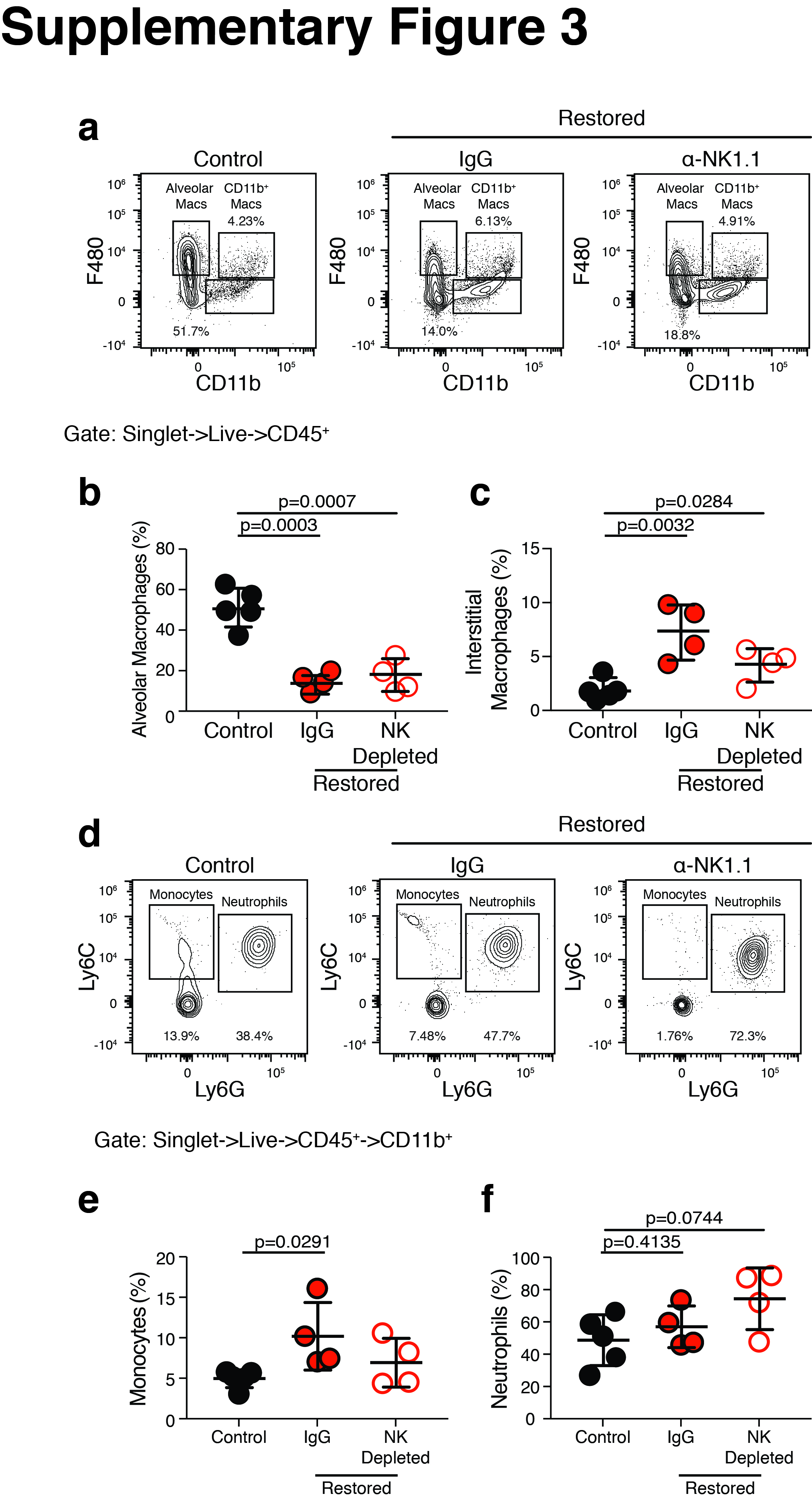

Supplement: Supplementary file 4 — Supplemental Figure 3 [file 41389_2019_133_MOESM4_ESM.jpg]

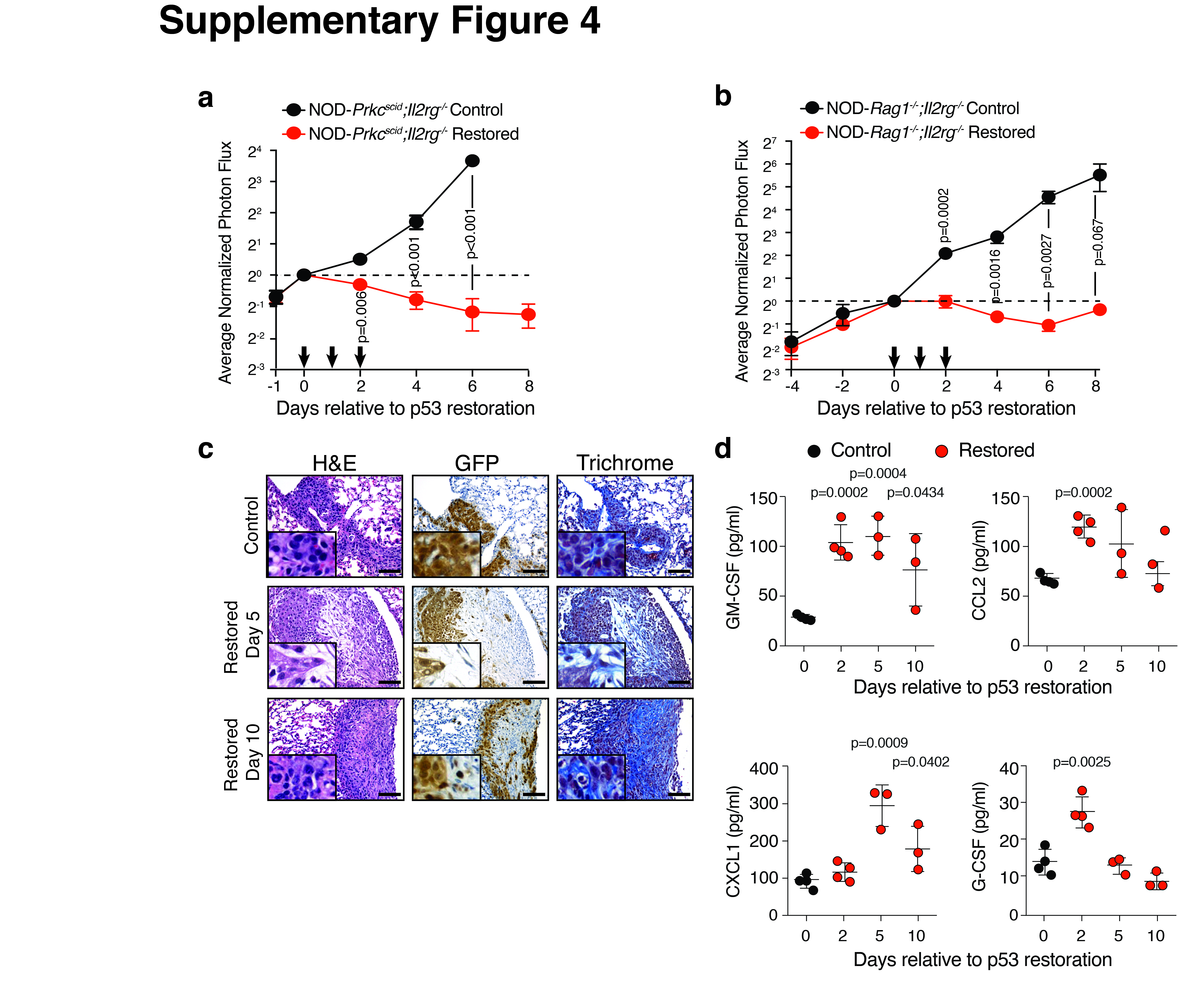

Supplement: Supplementary file 5 — Supplemental Figure 4 [file 41389_2019_133_MOESM5_ESM.jpg]
